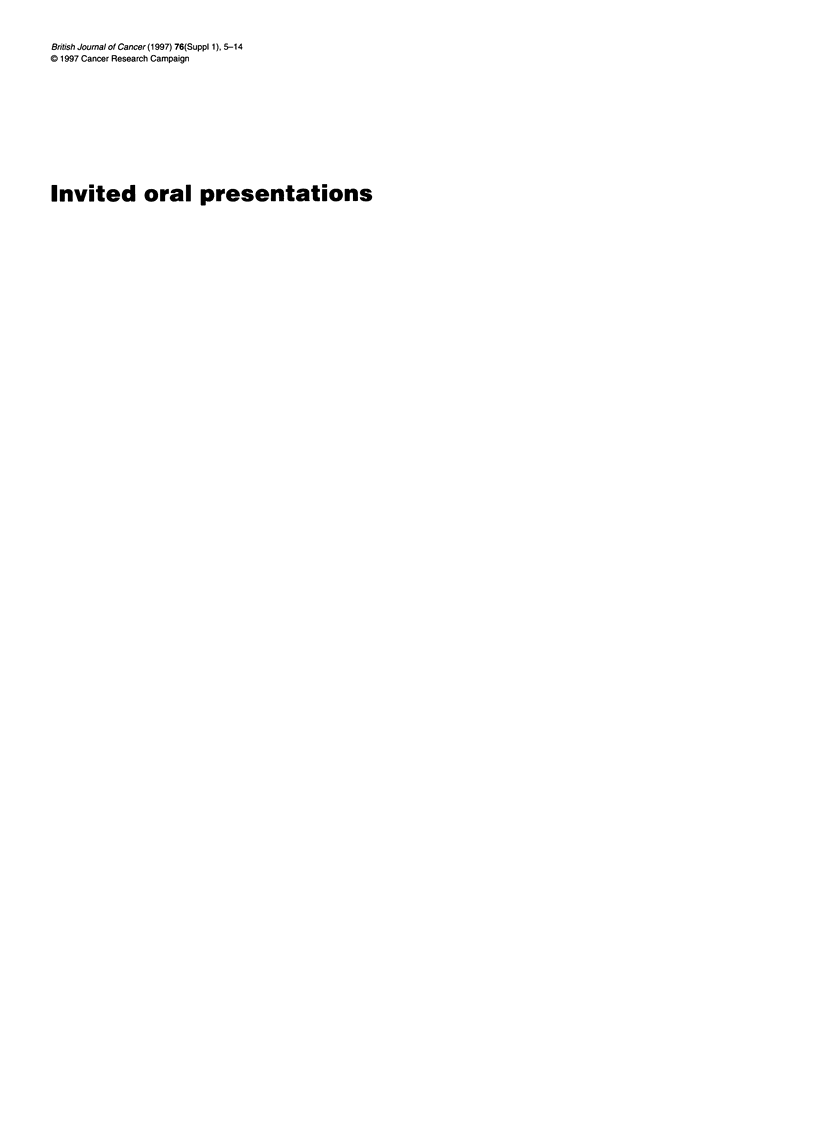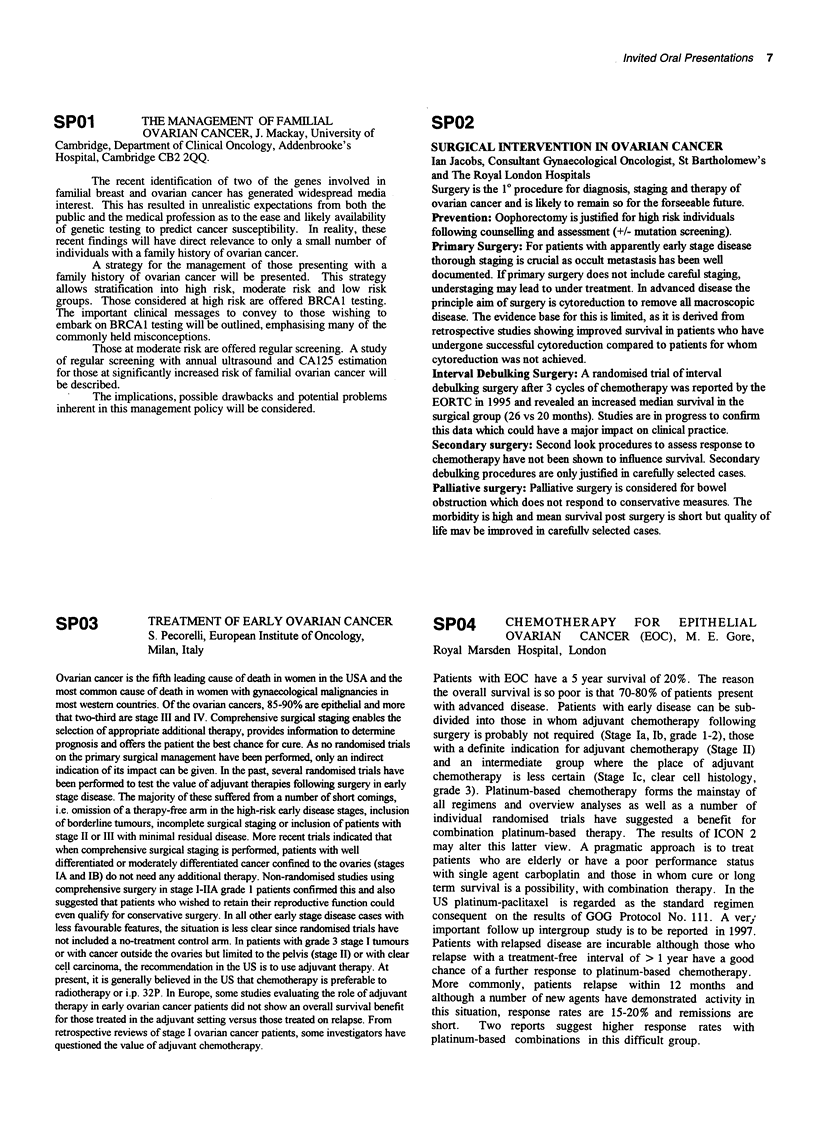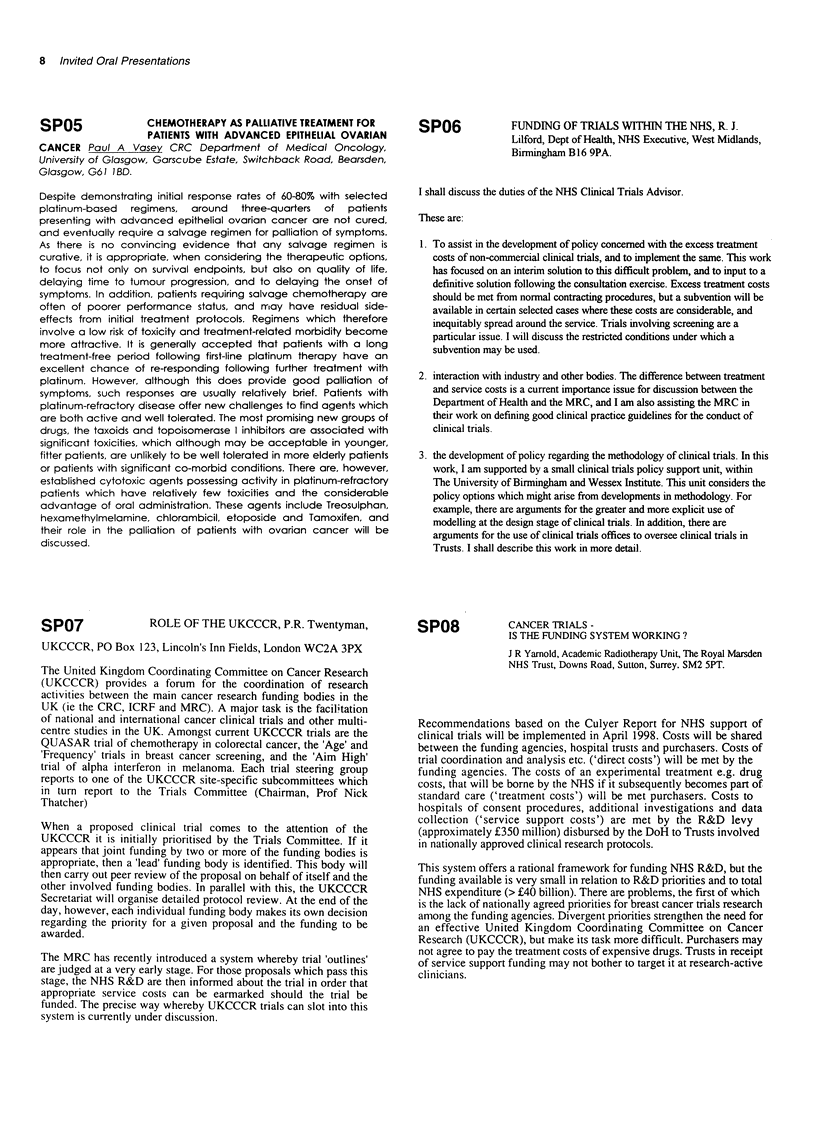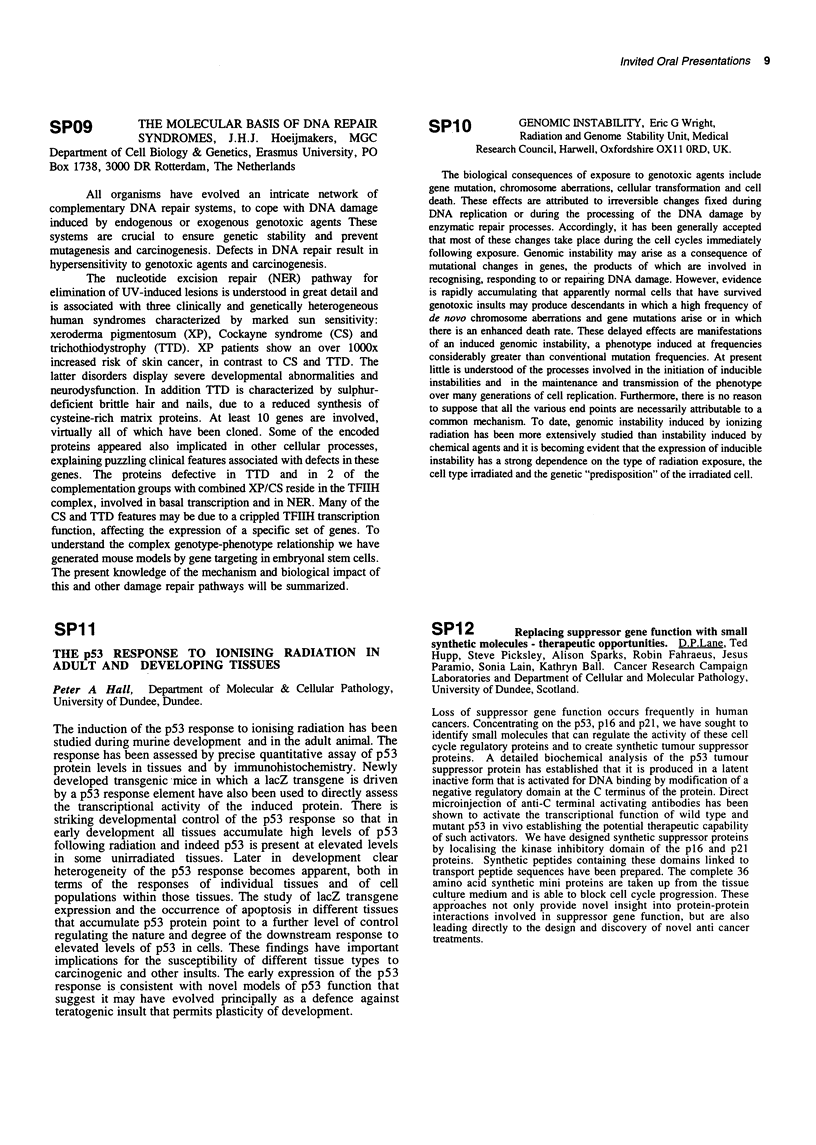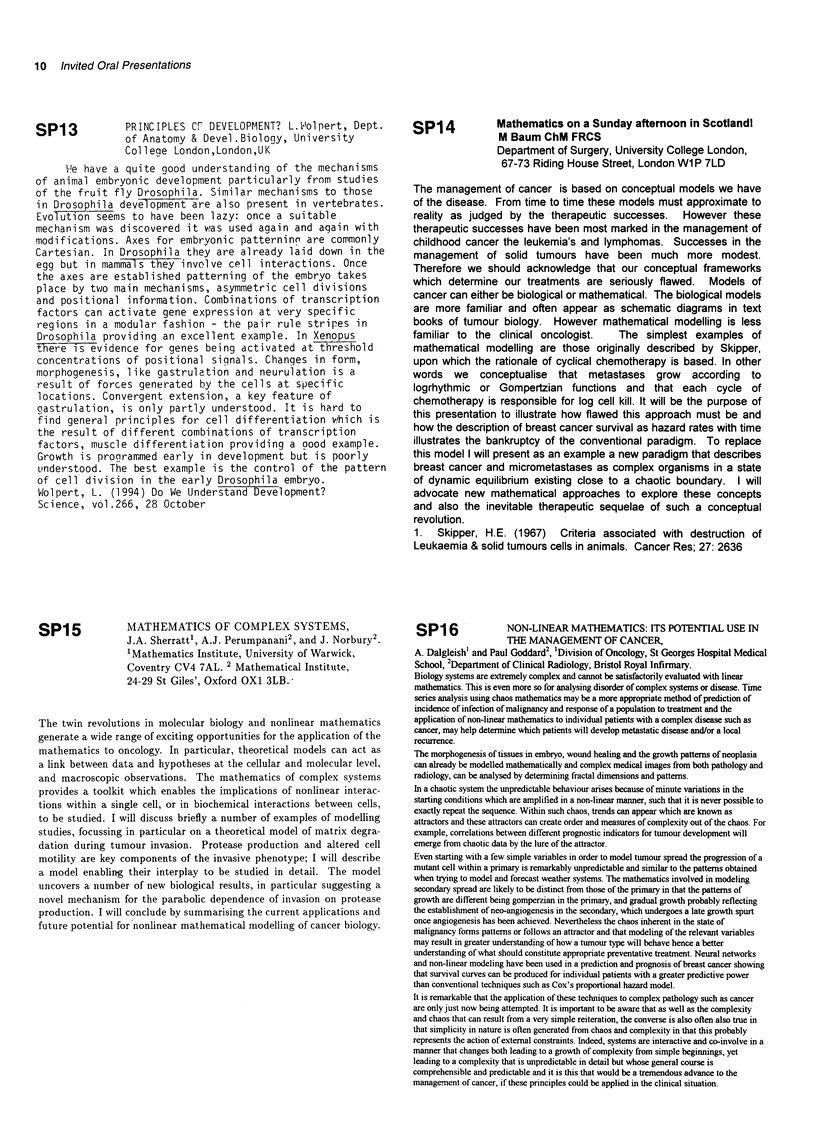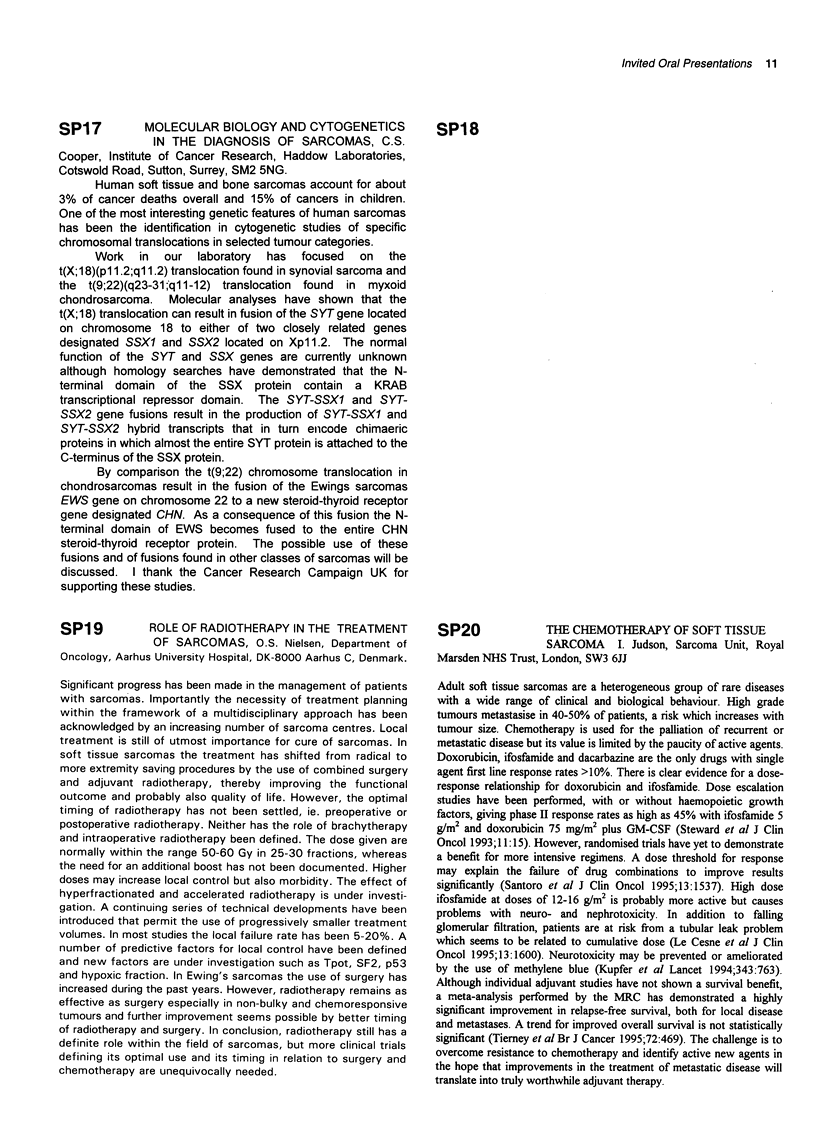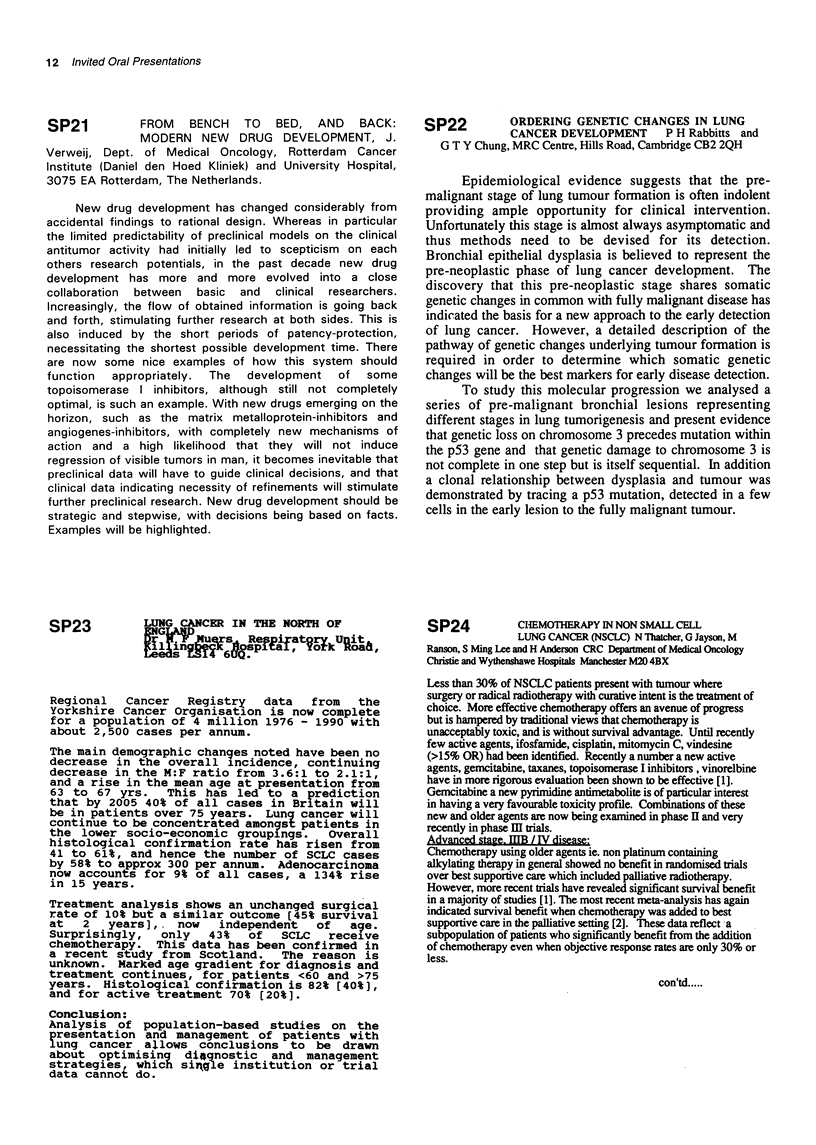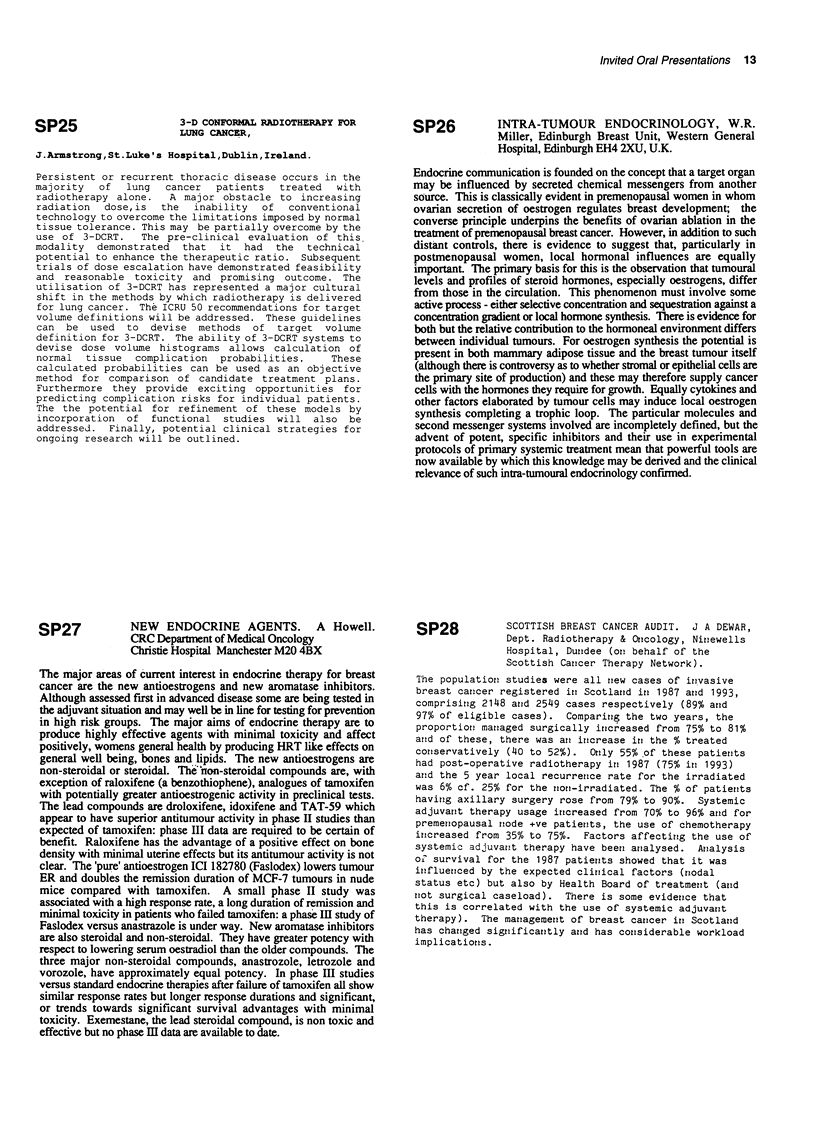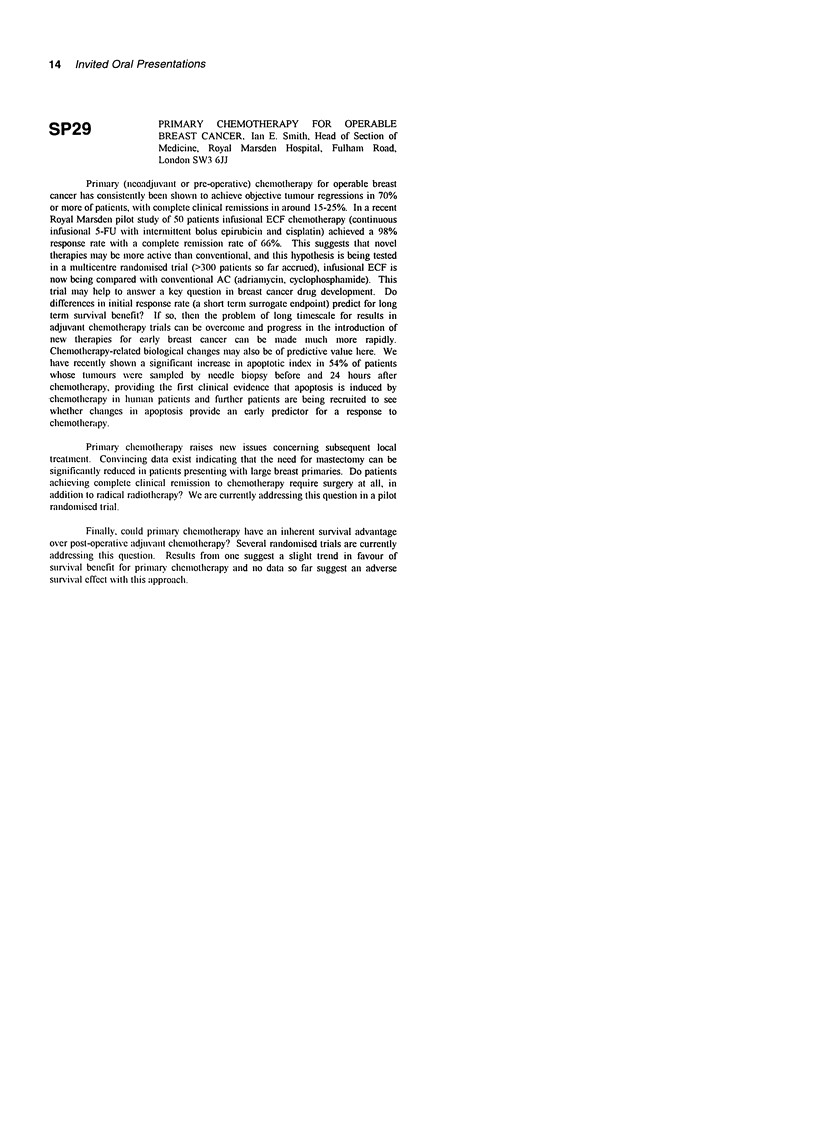# Invited oral presentations

**Published:** 1997

**Authors:** 


*British Journal of Cancer* (1997) **76**(Suppl 1), 5–14
© 1997 Cancer Research Campaign

### Invited oral presentations

### SP01

THE MANAGEMENT OF FAMILIAL OVARIAN CANCER, J. Mackay, University of Cambridge, Department of Clinical Oncology, Addenbrooke's Hospital, Cambridge CB2 2QQ.

The recent identification of two of the genes involved in familial breast and ovarian cancer has generated widespread media interest. This has resulted in unrealistic expectations from both the public and the medical profession as to the ease and likely availability of genetic testing to predict cancer susceptibility. In reality, these recent findings will have direct relevance to only a small number of individuals with a family history of ovarian cancer.

A strategy for the management of those presenting with a family history of ovarian cancer will be presented. This strategy allows stratification into high risk, moderate risk and low risk groups. Those considered at high risk are offered BRCA1 testing. The important clinical messages to convey to those wishing to embark on BRCA1 testing will be outlined, emphasising many of the commonly held misconceptions.

Those at moderate risk are offered regular screening. A study of regular screening with annual ultrasound and CA125 estimation for those at significantly increased risk of familial ovarian cancer will be described.

The implications, possible drawbacks and potential problems inherent in this management policy will be considered.

### SP02

**SURGICAL INTERVENTION IN OVARIAN CANCER**
**Ian Jacobs, Consultant Gynaecological Oncologist, St Bartholomew's and The Royal London Hospitals**

Surgery is the 1° procedure for diagnosis, staging and therapy of ovarian cancer and is likely to remain so for the forseeable future.
**Prevention:** Oophorectomy is justified for high risk individuals following counselling and assessment (+/- mutation screening).
**Primary Surgery:** For patients with apparently early stage disease thorough staging is crucial as occult metastasis has been well documented. If primary surgery does not include careful staging, understaging may lead to under treatment. In advanced disease the principle aim of surgery is cytoreduction to remove all macroscopic disease. The evidence base for this is limited, as it is derived from retrospective studies showing improved survival in patients who have undergone successful cytoreduction compared to patients for whom cytoreduction was not achieved.
**Interval Debulking Surgery:** A randomised trial of interval debulking surgery after 3 cycles of chemotherapy was reported by the EORTC in 1995 and revealed an increased median survival in the surgical group (26 vs 20 months). Studies are in progress to confirm this data which could have a major impact on clinical practice.
**Secondary surgery:** Second look procedures to assess response to chemotherapy have not been shown to influence survival. Secondary debulking procedures are only justified in carefully selected cases.
**Palliative surgery:** Palliative surgery is considered for bowel obstruction which does not respond to conservative measures. The morbidity is high and mean survival post surgery is short but quality of life mav be imoroved in carefullv selected cases.

### SP03

TREATMENT OF EARLY OVARIAN CANCER
S. Pecorelli, European Institute of Oncology, Milan, Italy

Ovarian cancer is the fifth leading cause of death in women in the USA and the most common cause of death in women with gynaecological malignancies in most western countries. Of the ovarian cancers, 85-90% are epithelial and more that two-third are stage III and IV. Comprehensive surgical staging enables the selection of appropriate additional therapy, provides information to determine prognosis and offers the patient the best chance for cure. As no randomised trials on the primary surgical management have been performed, only an indirect indication of its impact can be given. In the past, several randomised trials have been performed to test the value of adjuvant therapies following surgery in early stage disease. The majority of these suffered from a number of short comings, i.e. omission of a therapy-free arm in the high-risk early disease stages, inclusion of borderline tumours, incomplete surgical staging or inclusion of patients with stage II or III with minimal residual disease. More recent trials indicated that when comprehensive surgical staging is performed, patients with well differentiated or moderately differentiated cancer confined to the ovaries (stages IA and IB) do not need any additional therapy. Non-randomised studies using comprehensive surgery in stage I-IIA grade 1 patients confirmed this and also suggested that patients who wished to retain their reproductive function could even qualify for conservative surgery. In all other early stage disease cases with less favourable features, the situation is less clear since randomised trials have not included a no-treatment control arm. In patients with grade 3 stage I tumours or with cancer outside the ovaries but limited to the pelvis (stage II) or with clear cell carcinoma, the recommendation in the US is to use adjuvant therapy. At present, it is generally believed in the US that chemotherapy is preferable to radiotherapy or i.p. 32P. In Europe, some studies evaluating the role of adjuvant therapy in early ovarian cancer patients did not show an overall survival benefit for those treated in the adjuvant setting versus those treated on relapse. From retrospective reviews of stage I ovarian cancer patients, some investigators have questioned the value of adjuvant chemotherapy.

### SP04

CHEMOTHERAPY FOR EPITHELIAL OVARIAN CANCER (EOC), M. E. Gore, Royal Marsden Hospital, London

Patients with EOC have a 5 year survival of 20%. The reason the overall survival is so poor is that 70-80% of patients present with advanced disease. Patients with early disease can be subdivided into those in whom adjuvant chemotherapy following surgery is probably not required (Stage Ia, Ib, grade 1-2), those with a definite indication for adjuvant chemotherapy (Stage II) and an intermediate group where the place of adjuvant chemotherapy is less certain (Stage Ic, clear cell histology, grade 3). Platinum-based chemotherapy forms the mainstay of all regimens and overview analyses as well as a number of individual randomised trials have suggested a benefit for combination platinum-based therapy. The results of ICON 2 may alter this latter view. A pragmatic approach is to treat patients who are elderly or have a poor performance status with single agent carboplatin and those in whom cure or long term survival is a possibility, with combination therapy. In the US platinum-paclitaxel is regarded as the standard regimen consequent on the results of GOG Protocol No. 111. A very important follow up intergroup study is to be reported in 1997. Patients with relapsed disease are incurable although those who relapse with a treatment-free interval of > 1 year have a good chance of a further response to platinum-based chemotherapy. More commonly, patients relapse within 12 months and although a number of new agents have demonstrated activity in this situation, response rates are 15-20% and remissions are short. Two reports suggest higher response rates with platinum-based combinations in this difficult group.

### SP05

**CHEMOTHERAPY AS PALLIATIVE TREATMENT FOR PATIENTS WITH ADVANCED EPITHELIAL OVARIAN CANCER**

*Paul A Vasey CRC Department of Medical Oncology, University of Glasgow, Garscube Estate, Switchback Road, Bearsden, Glasgow, G61 1BD.*

Despite demonstrating initial response rates of 60-80% with selected platinum-based regimens, around three-quarters of patients presenting with advanced epithelial ovarian cancer are not cured, and eventually require a salvage regimen for palliation of symptoms. As there is no convincing evidence that any salvage regimen is curative, it is appropriate, when considering the therapeutic options, to focus not only on survival endpoints, but also on quality of life, delaying time to tumour progression, and to delaying the onset of symptoms. In addition, patients requiring salvage chemotherapy are often of poorer performance status, and may have residual side-effects from initial treatment protocols. Regimens which therefore involve a low risk of toxicity and treatment-related morbidity become more attractive. It is generally accepted that patients with a long treatment-free period following first-line platinum therapy have an excellent chance of re-responding following further treatment with platinum. However, although this does provide good palliation of symptoms, such responses are usually relatively brief. Patients with platinum-refractory disease offer new challenges to find agents which are both active and well tolerated. The most promising new groups of drugs, the taxoids and topoisomerase I inhibitors are associated with significant toxicities, which although may be acceptable in younger, fitter patients, are unlikely to be well tolerated in more elderly patients or patients with significant co-morbid conditions. There are, however, established cytotoxic agents possessing activity in platinum-refractory patients which have relatively few toxicities and the considerable advantage of oral administration. These agents include Treosulphan, hexamethylmelamine, chlorambicil, etoposide and Tamoxifen, and their role in the palliation of patients with ovarian cancer will be discussed.

### SP06

FUNDING OF TRIALS WITHIN THE NHS, R. J. Lilford, Dept of Health, NHS Executive, West Midlands, Birmingham B16 9PA.

I shall discuss the duties of the NHS Clinical Trials Advisor.

These are:

1. To assist in the development of policy concerned with the excess treatment costs of non-commercial clinical trials, and to implement the same. This work has focused on an interim solution to this difficult problem, and to input to a definitive solution following the consultation exercise. Excess treatment costs should be met from normal contracting procedures, but a subvention will be available in certain selected cases where these costs are considerable, and inequitably spread around the service. Trials involving screening are a particular issue. I will discuss the restricted conditions under which a subvention may be used.

2. interaction with industry and other bodies. The difference between treatment and service costs is a current importance issue for discussion between the Department of Health and the MRC, and I am also assisting the MRC in their work on defining good clinical practice guidelines for the conduct of clinical trials.

3. the development of policy regarding the methodology of clinical trials. In this work, I am supported by a small clinical trials policy support unit, within The University of Birmingham and Wessex Institute. This unit considers the policy options which might arise from developments in methodology. For example, there are arguments for the greater and more explicit use of modelling at the design stage of clinical trials. In addition, there are arguments for the use of clinical trials offices to oversee clinical trials in Trusts. I shall describe this work in more detail.

### SP07

ROLE OF THE UKCCCR, P.R. Twentyman, UKCCCR, PO Box 123, Lincoln's Inn Fields, London WC2A 3PX

The United Kingdom Coordinating Committee on Cancer Research (UKCCCR) provides a forum for the coordination of research activities between the main cancer research funding bodies in the UK (ie the CRC, ICRF and MRC). A major task is the facilitation of national and international cancer clinical trials and other multi-centre studies in the UK. Amongst current UKCCCR trials are the QUASAR trial of chemotherapy in colorectal cancer, the 'Age' and 'Frequency' trials in breast cancer screening, and the 'Aim High' trial of alpha interferon in melanoma. Each trial steering group reports to one of the UKCCCR site-specific subcommittees which in turn report to the Trials Committee (Chairman, Prof Nick Thatcher)

When a proposed clinical trial comes to the attention of the UKCCCR it is initially prioritised by the Trials Committee. If it appears that joint funding by two or more of the funding bodies is appropriate, then a 'lead' funding body is identified. This body will then carry out peer review of the proposal on behalf of itself and the other involved funding bodies. In parallel with this, the UKCCCR Secretariat will organise detailed protocol review. At the end of the day, however, each individual funding body makes its own decision regarding the priority for a given proposal and the funding to be awarded.

The MRC has recently introduced a system whereby trial 'outlines' are judged at a very early stage. For those proposals which pass this stage, the NHS R&D are then informed about the trial in order that appropriate service costs can be earmarked should the trial be funded. The precise way whereby UKCCCR trials can slot into this system is currently under discussion.

### SP08

CANCER TRIALS - IS THE FUNDING SYSTEM WORKING ?

J R Yarnold, Academic Radiotherapy Unit, The Royal Marsden NHS Trust, Downs Road, Sutton, Surrey. SM2 5PT.

Recommendations based on the Culyer Report for NHS support of clinical trials will be implemented in April 1998. Costs will be shared between the funding agencies, hospital trusts and purchasers. Costs of trial coordination and analysis etc. ('direct costs') will be met by the funding agencies. The costs of an experimental treatment e.g. drug costs, that will be borne by the NHS if it subsequently becomes part of standard care ('treatment costs') will be met purchasers. Costs to hospitals of consent procedures, additional investigations and data collection ('service support costs') are met by the R&D levy (approximately £350 million) disbursed by the DoH to Trusts involved in nationally approved clinical research protocols.

This system offers a rational framework for funding NHS R&D, but the funding available is very small in relation to R&D priorities and to total NHS expenditure (> £40 billion). There are problems, the first of which is the lack of nationally agreed priorities for breast cancer trials research among the funding agencies. Divergent priorities strengthen the need for an effective United Kingdom Coordinating Committee on Cancer Research (UKCCCR), but make its task more difficult. Purchasers may not agree to pay the treatment costs of expensive drugs. Trusts in receipt of service support funding may not bother to target it at research-active clinicians.

### SP09

THE MOLECULAR BASIS OF DNA REPAIR SYNDROMES, J.H.J. Hoeijmakers, MGC Department of Cell Biology & Genetics, Erasmus University, PO Box 1738, 3000 DR Rotterdam, The Netherlands

All organisms have evolved an intricate network of complementary DNA repair systems, to cope with DNA damage induced by endogenous or exogenous genotoxic agents These systems are crucial to ensure genetic stability and prevent mutagenesis and carcinogenesis. Defects in DNA repair result in hypersensitivity to genotoxic agents and carcinogenesis.

The nucleotide excision repair (NER) pathway for elimination of UV-induced lesions is understood in great detail and is associated with three clinically and genetically heterogeneous human syndromes characterized by marked sun sensitivity: xeroderma pigmentosum (XP), Cockayne syndrome (CS) and trichothiodystrophy (TTD). XP patients show an over 1000x increased risk of skin cancer, in contrast to CS and TTD. The latter disorders display severe developmental abnormalities and neurodysfunction. In addition TTD is characterized by sulphur-deficient brittle hair and nails, due to a reduced synthesis of cysteine-rich matrix proteins. At least 10 genes are involved, virtually all of which have been cloned. Some of the encoded proteins appeared also implicated in other cellular processes, explaining puzzling clinical features associated with defects in these genes. The proteins defective in TTD and in 2 of the complementation groups with combined XP/CS reside in the TFIIH complex, involved in basal transcription and in NER. Many of the CS and TTD features may be due to a crippled TFIIH transcription function, affecting the expression of a specific set of genes. To understand the complex genotype-phenotype relationship we have generated mouse models by gene targeting in embryonal stem cells. The present knowledge of the mechanism and biological impact of this and other damage repair pathways will be summarized.

### SP10

GENOMIC INSTABILITY, Eric G Wright, Radiation and Genome Stability Unit, Medical Research Council, Harwell, Oxfordshire OX11 0RD, UK.

The biological consequences of exposure to genotoxic agents include gene mutation, chromosome aberrations, cellular transformation and cell death. These effects are attributed to irreversible changes fixed during DNA replication or during the processing of the DNA damage by enzymatic repair processes. Accordingly, it has been generally accepted that most of these changes take place during the cell cycles immediately following exposure. Genomic instability may arise as a consequence of mutational changes in genes, the products of which are involved in recognising, responding to or repairing DNA damage. However, evidence is rapidly accumulating that apparently normal cells that have survived genotoxic insults may produce descendants in which a high frequency of *de novo* chromosome aberrations and gene mutations arise or in which there is an enhanced death rate. These delayed effects are manifestations of an induced genomic instability, a phenotype induced at frequencies considerably greater than conventional mutation frequencies. At present little is understood of the processes involved in the initiation of inducible instabilities and in the maintenance and transmission of the phenotype over many generations of cell replication. Furthermore, there is no reason to suppose that all the various end points are necessarily attributable to a common mechanism. To date, genomic instability induced by ionizing radiation has been more extensively studied than instability induced by chemical agents and it is becoming evident that the expression of inducible instability has a strong dependence on the type of radiation exposure, the cell type irradiated and the genetic "predisposition" of the irradiated cell.

### SP11

### THE p53 RESPONSE TO IONISING RADIATION IN ADULT AND DEVELOPING TISSUES

***Peter A Hall,*** Department of Molecular & Cellular Pathology, University of Dundee, Dundee.

The induction of the p53 response to ionising radiation has been studied during murine development and in the adult animal. The response has been assessed by precise quantitative assay of p53 protein levels in tissues and by immunohistochemistry. Newly developed transgenic mice in which a lacZ transgene is driven by a p53 response element have also been used to directly assess the transcriptional activity of the induced protein. There is striking developmental control of the p53 response so that in early development all tissues accumulate high levels of p53 following radiation and indeed p53 is present at elevated levels in some unirradiated tissues. Later in development clear heterogeneity of the p53 response becomes apparent, both in terms of the responses of individual tissues and of cell populations within those tissues. The study of lacZ transgene expression and the occurrence of apoptosis in different tissues that accumulate p53 protein point to a further level of control regulating the nature and degree of the downstream response to elevated levels of p53 in cells. These findings have important implications for the susceptibility of different tissue types to carcinogenic and other insults. The early expression of the p53 response is consistent with novel models of p53 function that suggest it may have evolved principally as a defence against teratogenic insult that permits plasticity of development.

### SP12

**Replacing suppressor gene function with small synthetic molecules - therapeutic opportunities.** D.P.Lane, Ted Hupp, Steve Picksley, Alison Sparks, Robin Fahraeus, Jesus Paramio, Sonia Lain, Kathryn Ball. Cancer Research Campaign Laboratories and Department of Cellular and Molecular Pathology, University of Dundee, Scotland.

Loss of suppressor gene function occurs frequently in human cancers. Concentrating on the p53, p16 and p21, we have sought to identify small molecules that can regulate the activity of these cell cycle regulatory proteins and to create synthetic tumour suppressor proteins. A detailed biochemical analysis of the p53 tumour suppressor protein has established that it is produced in a latent inactive form that is activated for DNA binding by modification of a negative regulatory domain at the C terminus of the protein. Direct microinjection of anti-C terminal activating antibodies has been shown to activate the transcriptional function of wild type and mutant p53 in vivo establishing the potential therapeutic capability of such activators. We have designed synthetic suppressor proteins by localising the kinase inhibitory domain of the p16 and p21 proteins. Synthetic peptides containing these domains linked to transport peptide sequences have been prepared. The complete 36 amino acid synthetic mini proteins are taken up from the tissue culture medium and is able to block cell cycle progression. These approaches not only provide novel insight into protein-protein interactions involved in suppressor gene function, but are also leading directly to the design and discovery of novel anti cancer treatments.

### SP13

PRINCIPLES OF DEVELOPMENT?
L.Wolpert, Dept. of Anatomy & Devel.Biology, University College London,London,UK

We have a quite good understanding of the mechanisms of animal embryonic development particularly from studies of the fruit fly *Drosophila*. Similar mechanisms to those in *Drosophila* development are also present in vertebrates. Evolution seems to have been lazy: once a suitable mechanism was discovered it was used again and again with modifications. Axes for embryonic patterning are commonly Cartesian. In *Drosophila* they are already laid down in the egg but in mammals they involve cell interactions. Once the axes are established patterning of the embryo takes place by two main mechanisms, asymmetric cell divisions and positional information. Combinations of transcription factors can activate gene expression at very specific regions in a modular fashion - the pair rule stripes in *Drosophila* providing an excellent example. In *Xenopus* there is evidence for genes being activated at threshold concentrations of positional signals. Changes in form, morphogenesis, like gastrulation and neurulation is a result of forces generated by the cells at specific locations. Convergent extension, a key feature of gastrulation, is only partly understood. It is hard to find general principles for cell differentiation which is the result of different combinations of transcription factors, muscle differentiation providing a good example. Growth is programmed early in development but is poorly understood. The best example is the control of the pattern of cell division in the early *Drosophila* embryo.

Wolpert, L. (1994) Do We Understand Development? Science, vol.266, 28 October

### SP14

**Mathematics on a Sunday afternoon in Scotland!**

**M Baum ChM FRCS**
Department of Surgery, University College London,
67-73 Riding House Street, London W1P 7LD

The management of cancer is based on conceptual models we have of the disease. From time to time these models must approximate to reality as judged by the therapeutic successes. However these therapeutic successes have been most marked in the management of childhood cancer the leukemia's and lymphomas. Successes in the management of solid tumours have been much more modest. Therefore we should acknowledge that our conceptual frameworks which determine our treatments are seriously flawed. Models of cancer can either be biological or mathematical. The biological models are more familiar and often appear as schematic diagrams in text books of tumour biology. However mathematical modelling is less familiar to the clinical oncologist. The simplest examples of mathematical modelling are those originally described by Skipper, upon which the rationale of cyclical chemotherapy is based. In other words we conceptualise that metastases grow according to logrhythmic or Gompertzian functions and that each cycle of chemotherapy is responsible for log cell kill. It will be the purpose of this presentation to illustrate how flawed this approach must be and how the description of breast cancer survival as hazard rates with time illustrates the bankruptcy of the conventional paradigm. To replace this model I will present as an example a new paradigm that describes breast cancer and micrometastases as complex organisms in a state of dynamic equilibrium existing close to a chaotic boundary. I will advocate new mathematical approaches to explore these concepts and also the inevitable therapeutic sequelae of such a conceptual revolution.

1. Skipper, H.E. (1967) Criteria associated with destruction of Leukaemia & solid tumours cells in animals. Cancer Res; 27: 2636

### SP15

MATHEMATICS OF COMPLEX SYSTEMS,

J.A. Sherratt[1], A.J. Perumpanani[2], and J. Norbury[2].
[1]Mathematics Institute, University of Warwick, Coventry CV4 7AL. [2] Mathematical Institute, 24-29 St Giles', Oxford OX1 3LB.

The twin revolutions in molecular biology and nonlinear mathematics generate a wide range of exciting opportunities for the application of the mathematics to oncology. In particular, theoretical models can act as a link between data and hypotheses at the cellular and molecular level, and macroscopic observations. The mathematics of complex systems provides a toolkit which enables the implications of nonlinear interactions within a single cell, or in biochemical interactions between cells, to be studied. I will discuss briefly a number of examples of modelling studies, focussing in particular on a theoretical model of matrix degradation during tumour invasion. Protease production and altered cell motility are key components of the invasive phenotype; I will describe a model enabling their interplay to be studied in detail. The model uncovers a number of new biological results, in particular suggesting a novel mechanism for the parabolic dependence of invasion on protease production. I will conclude by summarising the current applications and future potential for nonlinear mathematical modelling of cancer biology.

### SP16

NON-LINEAR MATHEMATICS: ITS POTENTIAL USE IN THE MANAGEMENT OF CANCER,

A. Dalgleish[1] and Paul Goddard[2], [1]Division of Oncology, St Georges Hospital Medical School, [2]Department of Clinical Radiology, Bristol Royal Infirmary.

Biology systems are extremely complex and cannot be satisfactorily evaluated with linear mathematics. This is even more so for analysing disorder of complex systems or disease. Time series analysis using chaos mathematics may be a more appropriate method of prediction of incidence of infection of malignancy and response of a population to treatment and the application of non-linear mathematics to individual patients with a complex disease such as cancer, may help determine which patients will develop metastatic disease and/or a local recurrence.

The morphogenesis of tissues in embryo, wound healing and the growth patterns of neoplasia can already be modelled mathematically and complex medical images from both pathology and radiology, can be analysed by determining fractal dimensions and patterns.

In a chaotic system the unpredictable behaviour arises because of minute variations in the starting conditions which are amplified in a non-linear manner, such that it is never possible to exactly repeat the sequence. Within such chaos, trends can appear which are known as attractors and these attractors can create order and measures of complexity out of the chaos. For example, correlations between different prognostic indicators for tumour development will emerge from chaotic data by the lure of the attractor.

Even starting with a few simple variables in order to model tumour spread the progression of a mutant cell within a primary is remarkably unpredictable and similar to the patterns obtained when trying to model and forecast weather systems. The mathematics involved in modeling secondary spread are likely to be distinct from those of the primary in that the patterns of growth are different being gomperzian in the primary, and gradual growth probably reflecting the establishment of neo-angiogenesis in the secondary, which undergoes a late growth spurt once angiogenesis has been achieved. Nevertheless the chaos inherent in the state of malignancy forms patterns or follows an attractor and that modeling of the relevant variables may result in greater understanding of how a tumour type will behave hence a better understanding of what should constitute appropriate preventative treatment. Neural networks and non-linear modeling have been used in a prediction and prognosis of breast cancer showing that survival curves can be produced for individual patients with a greater predictive power than conventional techniques such as Cox's proportional hazard model.

It is remarkable that the application of these techniques to complex pathology such as cancer are only just now being attempted. It is important to be aware that as well as the complexity and chaos that can result from a very simple reiteration, the converse is also often also true in that simplicity in nature is often generated from chaos and complexity in that this probably represents the action of external constraints. Indeed, systems are interactive and co-involve in a manner that changes both leading to a growth of complexity from simple beginnings, yet leading to a complexity that is unpredictable in detail but whose general course is comprehensible and predictable and it is this that would be a tremendous advance to the management of cancer, if these principles could be applied in the clinical situation.

### SP17

MOLECULAR BIOLOGY AND CYTOGENETICS IN THE DIAGNOSIS OF SARCOMAS, C.S. Cooper, Institute of Cancer Research, Haddow Laboratories, Cotswold Road, Sutton, Surrey, SM2 5NG.

Human soft tissue and bone sarcomas account for about 3% of cancer deaths overall and 15% of cancers in children. One of the most interesting genetic features of human sarcomas has been the identification in cytogenetic studies of specific chromosomal translocations in selected tumour categories.

Work in our laboratory has focused on the t(X;18)(p11.2;q11.2) translocation found in synovial sarcoma and the t(9;22)(q23-31;q11-12) translocation found in myxoid chondrosarcoma. Molecular analyses have shown that the t(X;18) translocation can result in fusion of the *SYT* gene located on chromosome 18 to either of two closely related genes designated *SSX1* and *SSX2* located on Xp11.2. The normal function of the *SYT* and *SSX* genes are currently unknown although homology searches have demonstrated that the N-terminal domain of the SSX protein contain a KRAB transcriptional repressor domain. The *SYT-SSX1* and *SYT-SSX2* gene fusions result in the production of *SYT-SSX1* and *SYT-SSX2* hybrid transcripts that in turn encode chimaeric proteins in which almost the entire SYT protein is attached to the C-terminus of the SSX protein.

By comparison the t(9;22) chromosome translocation in chondrosarcomas result in the fusion of the Ewings sarcomas *EWS* gene on chromosome 22 to a new steroid-thyroid receptor gene designated *CHN*. As a consequence of this fusion the N-terminal domain of EWS becomes fused to the entire CHN steroid-thyroid receptor protein. The possible use of these fusions and of fusions found in other classes of sarcomas will be discussed. I thank the Cancer Research Campaign UK for supporting these studies.

### SP18

### SP19

ROLE OF RADIOTHERAPY IN THE TREATMENT OF SARCOMAS, O.S. Nielsen, Department of Oncology, Aarhus University Hospital, DK-8000 Aarhus C, Denmark.

Significant progress has been made in the management of patients with sarcomas. Importantly the necessity of treatment planning within the framework of a multidisciplinary approach has been acknowledged by an increasing number of sarcoma centres. Local treatment is still of utmost importance for cure of sarcomas. In soft tissue sarcomas the treatment has shifted from radical to more extremity saving procedures by the use of combined surgery and adjuvant radiotherapy, thereby improving the functional outcome and probably also quality of life. However, the optimal timing of radiotherapy has not been settled, ie. preoperative or postoperative radiotherapy. Neither has the role of brachytherapy and intraoperative radiotherapy been defined. The dose given are normally within the range 50-60 Gy in 25-30 fractions, whereas the need for an additional boost has not been documented. Higher doses may increase local control but also morbidity. The effect of hyperfractionated and accelerated radiotherapy is under investigation. A continuing series of technical developments have been introduced that permit the use of progressively smaller treatment volumes. In most studies the local failure rate has been 5-20%. A number of predictive factors for local control have been defined and new factors are under investigation such as Tpot, SF2, p53 and hypoxic fraction. In Ewing's sarcomas the use of surgery has increased during the past years. However, radiotherapy remains as effective as surgery especially in non-bulky and chemoresponsive tumours and further improvement seems possible by better timing of radiotherapy and surgery. In conclusion, radiotherapy still has a definite role within the field of sarcomas, but more clinical trials defining its optimal use and its timing in relation to surgery and chemotherapy are unequivocally needed.

### SP20

THE CHEMOTHERAPY OF SOFT TISSUE SARCOMA I. Judson, Sarcoma Unit, Royal Marsden NHS Trust, London, SW3 6JJ

Adult soft tissue sarcomas are a heterogeneous group of rare diseases with a wide range of clinical and biological behaviour. High grade tumours metastasise in 40-50% of patients, a risk which increases with tumour size. Chemotherapy is used for the palliation of recurrent or metastatic disease but its value is limited by the paucity of active agents. Doxorubicin, ifosfamide and dacarbazine are the only drugs with single agent first line response rates >10%. There is clear evidence for a dose-response relationship for doxorubicin and ifosfamide. Dose escalation studies have been performed, with or without haemopoietic growth factors, giving phase II response rates as high as 45% with ifosfamide 5 $g/m^2$ and doxorubicin 75 $mg/m^2$ plus GM-CSF (Steward *et al* J Clin Oncol 1993;11:15). However, randomised trials have yet to demonstrate a benefit for more intensive regimens. A dose threshold for response may explain the failure of drug combinations to improve results significantly (Santoro *et al* J Clin Oncol 1995;13:1537). High dose ifosfamide at doses of 12-16 $g/m^2$ is probably more active but causes problems with neuro- and nephrotoxicity. In addition to falling glomerular filtration, patients are at risk from a tubular leak problem which seems to be related to cumulative dose (Le Cesne *et al* J Clin Oncol 1995;13:1600). Neurotoxicity may be prevented or ameliorated by the use of methylene blue (Kupfer *et al* Lancet 1994;343:763). Although individual adjuvant studies have not shown a survival benefit, a meta-analysis performed by the MRC has demonstrated a highly significant improvement in relapse-free survival, both for local disease and metastases. A trend for improved overall survival is not statistically significant (Tierney *et al* Br J Cancer 1995;72:469). The challenge is to overcome resistance to chemotherapy and identify active new agents in the hope that improvements in the treatment of metastatic disease will translate into truly worthwhile adjuvant therapy.

### SP21 FROM BENCH TO BED, AND BACK: MODERN NEW DRUG DEVELOPMENT

J. Verweij, Dept. of Medical Oncology, Rotterdam Cancer Institute (Daniel den Hoed Kliniek) and University Hospital, 3075 EA Rotterdam, The Netherlands.

New drug development has changed considerably from accidental findings to rational design. Whereas in particular the limited predictability of preclinical models on the clinical antitumor activity had initially led to scepticism on each others research potentials, in the past decade new drug development has more and more evolved into a close collaboration between basic and clinical researchers. Increasingly, the flow of obtained information is going back and forth, stimulating further research at both sides. This is also induced by the short periods of patency-protection, necessitating the shortest possible development time. There are now some nice examples of how this system should function appropriately. The development of some topoisomerase I inhibitors, although still not completely optimal, is such an example. With new drugs emerging on the horizon, such as the matrix metalloprotein-inhibitors and angiogenes-inhibitors, with completely new mechanisms of action and a high likelihood that they will not induce regression of visible tumors in man, it becomes inevitable that preclinical data will have to guide clinical decisions, and that clinical data indicating necessity of refinements will stimulate further preclinical research. New drug development should be strategic and stepwise, with decisions being based on facts. Examples will be highlighted.

### SP22 ORDERING GENETIC CHANGES IN LUNG CANCER DEVELOPMENT

P H Rabbitts and G T Y Chung, MRC Centre, Hills Road, Cambridge CB2 2QH

Epidemiological evidence suggests that the pre-malignant stage of lung tumour formation is often indolent providing ample opportunity for clinical intervention. Unfortunately this stage is almost always asymptomatic and thus methods need to be devised for its detection. Bronchial epithelial dysplasia is believed to represent the pre-neoplastic phase of lung cancer development. The discovery that this pre-neoplastic stage shares somatic genetic changes in common with fully malignant disease has indicated the basis for a new approach to the early detection of lung cancer. However, a detailed description of the pathway of genetic changes underlying tumour formation is required in order to determine which somatic genetic changes will be the best markers for early disease detection.

To study this molecular progression we analysed a series of pre-malignant bronchial lesions representing different stages in lung tumorigenesis and present evidence that genetic loss on chromosome 3 precedes mutation within the p53 gene and that genetic damage to chromosome 3 is not complete in one step but is itself sequential. In addition a clonal relationship between dysplasia and tumour was demonstrated by tracing a p53 mutation, detected in a few cells in the early lesion to the fully malignant tumour.

### SP23 LUNG CANCER IN THE NORTH OF ENGLAND

Dr M F Muers, Respiratory Unit, Killingbeck Hospital, York Road, Leeds LS14 6UQ.

Regional Cancer Registry data from the Yorkshire Cancer Organisation is now complete for a population of 4 million 1976 - 1990 with about 2,500 cases per annum.

The main demographic changes noted have been no decrease in the overall incidence, continuing decrease in the M:F ratio from 3.6:1 to 2.1:1, and a rise in the mean age at presentation from 63 to 67 yrs. This has led to a prediction that by 2005 40% of all cases in Britain will be in patients over 75 years. Lung cancer will continue to be concentrated amongst patients in the lower socio-economic groupings. Overall histological confirmation rate has risen from 41 to 61%, and hence the number of SCLC cases by 58% to approx 300 per annum. Adenocarcinoma now accounts for 9% of all cases, a 134% rise in 15 years.

Treatment analysis shows an unchanged surgical rate of 10% but a similar outcome [45% survival at 2 years], now independent of age. Surprisingly, only 43% of SCLC receive chemotherapy. This data has been confirmed in a recent study from Scotland. The reason is unknown. Marked age gradient for diagnosis and treatment continues, for patients <60 and >75 years. Histological confirmation is 82% [40%], and for active treatment 70% [20%].

**Conclusion:**
Analysis of population-based studies on the presentation and management of patients with lung cancer allows conclusions to be drawn about optimising diagnostic and management strategies, which single institution or trial data cannot do.

### SP24 CHEMOTHERAPY IN NON SMALL CELL LUNG CANCER (NSCLC)

N Thatcher, G Jayson, M Ranson, S Ming Lee and H Anderson CRC Department of Medical Oncology Christie and Wythenshawe Hospitals Manchester M20 4BX

Less than 30% of NSCLC patients present with tumour where surgery or radical radiotherapy with curative intent is the treatment of choice. More effective chemotherapy offers an avenue of progress but is hampered by traditional views that chemotherapy is unacceptably toxic, and is without survival advantage. Until recently few active agents, ifosfamide, cisplatin, mitomycin C, vindesine (>15% OR) had been identified. Recently a number a new active agents, gemcitabine, taxanes, topoisomerase I inhibitors , vinorelbine have in more rigorous evaluation been shown to be effective [1]. Gemcitabine a new pyrimidine antimetabolite is of particular interest in having a very favourable toxicity profile. Combinations of these new and older agents are now being examined in phase II and very recently in phase III trials.

Advanced stage, IIIB / IV disease:
Chemotherapy using older agents ie. non platinum containing alkylating therapy in general showed no benefit in randomised trials over best supportive care which included palliative radiotherapy. However, more recent trials have revealed significant survival benefit in a majority of studies [1]. The most recent meta-analysis has again indicated survival benefit when chemotherapy was added to best supportive care in the palliative setting [2]. These data reflect a subpopulation of patients who significantly benefit from the addition of chemotherapy even when objective response rates are only 30% or less.

con'td.....

### SP25 3-D CONFORMAL RADIOTHERAPY FOR LUNG CANCER,

**J.Armstrong,St.Luke's Hospital,Dublin,Ireland.**

Persistent or recurrent thoracic disease occurs in the majority of lung cancer patients treated with radiotherapy alone. A major obstacle to increasing radiation dose,is the inability of conventional technology to overcome the limitations imposed by normal tissue tolerance. This may be partially overcome by the use of 3-DCRT. The pre-clinical evaluation of this modality demonstrated that it had the technical potential to enhance the therapeutic ratio. Subsequent trials of dose escalation have demonstrated feasibility and reasonable toxicity and promising outcome. The utilisation of 3-DCRT has represented a major cultural shift in the methods by which radiotherapy is delivered for lung cancer. The ICRU 50 recommendations for target volume definitions will be addressed. These guidelines can be used to devise methods of target volume definition for 3-DCRT. The ability of 3-DCRT systems to devise dose volume histograms allows calculation of normal tissue complication probabilities. These calculated probabilities can be used as an objective method for comparison of candidate treatment plans. Furthermore they provide exciting opportunities for predicting complication risks for individual patients. The the potential for refinement of these models by incorporation of functional studies will also be addressed. Finally, potential clinical strategies for ongoing research will be outlined.

### SP26 INTRA-TUMOUR ENDOCRINOLOGY,

W.R. Miller, Edinburgh Breast Unit, Western General Hospital, Edinburgh EH4 2XU, U.K.

Endocrine communication is founded on the concept that a target organ may be influenced by secreted chemical messengers from another source. This is classically evident in premenopausal women in whom ovarian secretion of oestrogen regulates breast development; the converse principle underpins the benefits of ovarian ablation in the treatment of premenopausal breast cancer. However, in addition to such distant controls, there is evidence to suggest that, particularly in postmenopausal women, local hormonal influences are equally important. The primary basis for this is the observation that tumoural levels and profiles of steroid hormones, especially oestrogens, differ from those in the circulation. This phenomenon must involve some active process - either selective concentration and sequestration against a concentration gradient or local hormone synthesis. There is evidence for both but the relative contribution to the hormoneal environment differs between individual tumours. For oestrogen synthesis the potential is present in both mammary adipose tissue and the breast tumour itself (although there is controversy as to whether stromal or epithelial cells are the primary site of production) and these may therefore supply cancer cells with the hormones they require for growth. Equally cytokines and other factors elaborated by tumour cells may induce local oestrogen synthesis completing a trophic loop. The particular molecules and second messenger systems involved are incompletely defined, but the advent of potent, specific inhibitors and their use in experimental protocols of primary systemic treatment mean that powerful tools are now available by which this knowledge may be derived and the clinical relevance of such intra-tumoural endocrinology confirmed.

### SP27 NEW ENDOCRINE AGENTS.

A Howell. CRC Department of Medical Oncology Christie Hospital Manchester M20 4BX

The major areas of current interest in endocrine therapy for breast cancer are the new antioestrogens and new aromatase inhibitors. Although assessed first in advanced disease some are being tested in the adjuvant situation and may well be in line for testing for prevention in high risk groups. The major aims of endocrine therapy are to produce highly effective agents with minimal toxicity and affect positively, womens general health by producing HRT like effects on general well being, bones and lipids. The new antioestrogens are non-steroidal or steroidal. The non-steroidal compounds are, with exception of raloxifene (a benzothiophene), analogues of tamoxifen with potentially greater antioestrogenic activity in preclinical tests. The lead compounds are droloxifene, idoxifene and TAT-59 which appear to have superior antitumour activity in phase II studies than expected of tamoxifen: phase III data are required to be certain of benefit. Raloxifene has the advantage of a positive effect on bone density with minimal uterine effects but its antitumour activity is not clear. The 'pure' antioestrogen ICI 182780 (Faslodex) lowers tumour ER and doubles the remission duration of MCF-7 tumours in nude mice compared with tamoxifen. A small phase II study was associated with a high response rate, a long duration of remission and minimal toxicity in patients who failed tamoxifen: a phase III study of Faslodex versus anastrazole is under way. New aromatase inhibitors are also steroidal and non-steroidal. They have greater potency with respect to lowering serum oestradiol than the older compounds. The three major non-steroidal compounds, anastrozole, letrozole and vorozole, have approximately equal potency. In phase III studies versus standard endocrine therapies after failure of tamoxifen all show similar response rates but longer response durations and significant, or trends towards significant survival advantages with minimal toxicity. Exemestane, the lead steroidal compound, is non toxic and effective but no phase III data are available to date.

### SP28 SCOTTISH BREAST CANCER AUDIT.

J A DEWAR, Dept. Radiotherapy & Oncology, Ninewells Hospital, Dundee (on behalf of the Scottish Cancer Therapy Network).

The population studies were all new cases of invasive breast cancer registered in Scotland in 1987 and 1993, comprising 2148 and 2549 cases respectively (89% and 97% of eligible cases). Comparing the two years, the proportion managed surgically increased from 75% to 81% and of these, there was an increase in the % treated conservatively (40 to 52%). Only 55% of these patients had post-operative radiotherapy in 1987 (75% in 1993) and the 5 year local recurrence rate for the irradiated was 6% cf. 25% for the non-irradiated. The % of patients having axillary surgery rose from 79% to 90%. Systemic adjuvant therapy usage increased from 70% to 96% and for premenopausal node +ve patients, the use of chemotherapy increased from 35% to 75%. Factors affecting the use of systemic adjuvant therapy have been analysed. Analysis of survival for the 1987 patients showed that it was influenced by the expected clinical factors (nodal status etc) but also by Health Board of treatment (and not surgical caseload). There is some evidence that this is correlated with the use of systemic adjuvant therapy). The management of breast cancer in Scotland has changed significantly and has considerable workload implications.

### SP29

PRIMARY CHEMOTHERAPY FOR OPERABLE BREAST CANCER. Ian E. Smith, Head of Section of Medicine, Royal Marsden Hospital, Fulham Road, London SW3 6JJ

Primary (neoadjuvant or pre-operative) chemotherapy for operable breast cancer has consistently been shown to achieve objective tumour regressions in 70% or more of patients, with complete clinical remissions in around 15-25%. In a recent Royal Marsden pilot study of 50 patients infusional ECF chemotherapy (continuous infusional 5-FU with intermittent bolus epirubicin and cisplatin) achieved a 98% response rate with a complete remission rate of 66%. This suggests that novel therapies may be more active than conventional, and this hypothesis is being tested in a multicentre randomised trial (>300 patients so far accrued), infusional ECF is now being compared with conventional AC (adriamycin, cyclophosphamide). This trial may help to answer a key question in breast cancer drug development. Do differences in initial response rate (a short term surrogate endpoint) predict for long term survival benefit? If so, then the problem of long timescale for results in adjuvant chemotherapy trials can be overcome and progress in the introduction of new therapies for early breast cancer can be made much more rapidly. Chemotherapy-related biological changes may also be of predictive value here. We have recently shown a significant increase in apoptotic index in 54% of patients whose tumours were sampled by needle biopsy before and 24 hours after chemotherapy, providing the first clinical evidence that apoptosis is induced by chemotherapy in human patients and further patients are being recruited to see whether changes in apoptosis provide an early predictor for a response to chemotherapy.

Primary chemotherapy raises new issues concerning subsequent local treatment. Convincing data exist indicating that the need for mastectomy can be significantly reduced in patients presenting with large breast primaries. Do patients achieving complete clinical remission to chemotherapy require surgery at all, in addition to radical radiotherapy? We are currently addressing this question in a pilot randomised trial.

Finally, could primary chemotherapy have an inherent survival advantage over post-operative adjuvant chemotherapy? Several randomised trials are currently addressing this question. Results from one suggest a slight trend in favour of survival benefit for primary chemotherapy and no data so far suggest an adverse survival effect with this approach.